# Xenointoxication of a Rabbit for the Control of the Common Bed Bug *Cimex lectularius* L. Using Ivermectin

**DOI:** 10.1155/2019/4793569

**Published:** 2019-02-27

**Authors:** Gale E. Ridge, Wade Elmer, Stephanie Gaines, Xiaolin Li, Danie Schlatzer, Kim McClure-Brinton, Johnathan M. Sheele

**Affiliations:** ^1^The Connecticut Agricultural Experiment Station, 123 Huntington Street, P.O. Box 1106, New Haven, CT 06504, USA; ^2^University Hospitals Cleveland Medical Center & Case Western Reserve University, Department of Emergency Medicine, 11100 Euclid Ave., Cleveland, OH 44106, USA; ^3^Case Western Reserve University, 11000 Euclid Ave., Cleveland, OH 44106, USA; ^4^Country Companions Veterinary Services, 116 Old Amity Road, Bethany, CT 06524, USA

## Abstract

Human bed bug infestations have undergone a recent global resurgence. The human antiparasitic drug ivermectin has been proposed as a strategy to help control bed bug infestations, but *in vivo* data are lacking. We allowed separate populations of the common bed bug, *Cimex lectularius* L., to feed once on a rabbit before and after it was injected subcutaneously with 0.3 mg/kg of ivermectin, and bed bug morbidity and mortality were recorded. Ivermectin levels in the rabbit were measured using high-performance liquid chromatography and mass spectroscopy. Ivermectin blood levels of ∼2 ng/mL caused reductions in bed bug fecundity, and levels of >8 ng/mL caused bed bug death and long-term morbidity including reductions in refeeding, mobility, reproduction, and molting. Gut bacterial cultures from the fed bed bugs showed that ivermectin altered the bed bug gut microbiome.

## 1. Introduction

The common bed bug, *Cimex lectularius* Linnaeus (1758) is a cosmopolitan anthropophilic hematophagous temporary human ectoparasite [1, 2]. *Cimex lectularius* has made a recent global resurgence, and current bed bug control is significantly hampered by an increasing resistance of the insect to pesticides used in their control [3–6]. Bed bugs are arguably now one of the most important human ectoparasites in western industrialized nations, and new approaches are needed for managing infestations.

The pharmaceutical drug ivermectin is one of the most common antiparasitic drugs given to human, and is used to treat many human ectoparasites including *Pediculus humanus capitis* (head lice)*, Pediculus humanus humanus* (body lice), *Pthirus pubis* (pubic lice), and *Sarcoptes scabiei* (scabies). Ivermectin is not currently used in the control of *C. lectularius*, but ivermectin has been shown to kill bed bugs at plasma levels that have been reported in humans taking the drug [7–11]. Ivermectin binds to the invertebrate glutamate-gated chloride channel causing cellular hyperpolarization leading to paralysis and death [12].

The existing research involving ivermectin and bed bugs has focused on *in vitro* feedings where blood samples are spiked with ivermectin and fed to bed bugs in an artificial feeding system [9, 10]. These *in vitro* conditions likely do not replicate *in vivo* conditions principally for two reasons: pharmacokinetic studies of ivermectin in humans report ivermectin levels as plasma concentration, but bed bugs consume whole blood meals, and the secondary metabolites of ivermectin likely have antiparasitic effects that may persist beyond the parent compound. Ivermectin added to defibrinated *in vitro* blood samples will not undergo appreciable metabolism, so any secondary metabolites with toxic effects against bed bugs cannot be studied. Any ivermectin that accumulates intracellularly in hematogenous cells under *in vivo* conditions would not be measured in the plasma concentration. The *in vivo* concentration of ivermectin in the blood that is required to cause bed bug toxicity is unknown.

The objective of our study was to record bed bug mortality and the insects interest and ability to refeed, fecundity, activity level, and molting after the insects fed on a rabbit injected with ivermectin. Our secondary objectives were to correlate blood ivermectin levels in the rabbit with bed bug morbidity and mortality and to determine if ivermectin affects the bed bug gut microbiome.

## 2. Materials and Methods

### 2.1. Rabbit

We received Institutional Animal Care and Use Committee's (IACUC) approval. We used a two-year-old New Zealand white neutered male rabbit weighing 4.54 kg. Ivermectin (1% sterile solution) (Noromectin®, Norbrook Laboratories, UK) at 0.3 mg/kg was injected subcutaneously into the left shoulder of the rabbit by a veterinarian. The rabbit was trained to sit in the lap of a handler while the insects fed on a shaved left hip. The same rabbit was used for both experiments which were separated by more than a month.

### 2.2. Insects

Ridge-strain *C. lectularius* L. was used in the experiments. These insects were first collected in a New Haven, Connecticut, apartment in 2009 and have been maintained under laboratory conditions by feeding on a human volunteer. Populations were kept in small glass canning jars with open mouths covered by sheer fabric secured by a metal band. Inside each jar were vertical cardboard wafers where insects established natural refuges. Insects were exposed to natural daylight cycles at a temperature of ∼24°C (75°F) and 40–50% RH conditions.

Featherweight surface forceps were used to move insects. Twelve populations of five males, five females, and 10 mixed age instar nymphs were placed into 55 × 25 mm (4 dram) glass vials which contained a single piece of 10 × 40 mm card for perching. To keep stress low, vials were populated with 20 fully sated insects seven days before each test and left undisturbed. A 60 × 60 mm square of sheer fabric was secured over the vial mouth with an elastic band. When inverted, the card slid to the vial mouth, allowing insects to feed through the fabric.

### 2.3. Feeding Experiment

Populations of 5 females, 5 males, and 10 nymphs fed once on the rabbit at specific postinjection time intervals 0 (control—just before the rabbit was given ivermectin), 1, 6, 12, 24, 36, 48, 72, 96, 120, 144, and 168 hours were taken. All bed bugs fed to repletion. The experiment was repeated twice (Test 1 and Test 2).

Test 1 recorded the health of the bed bugs up to day 45 after feeding. Test 2 recorded the health of the bed bug up to day 64 after feeding but also included a breath test on day 14—performed by gently exhaling onto the insects and observing levels of stimulation and opportunities to feed on an investigator at days 16 and 31. Bed bugs were categorized as healthy (alert and active); reactive (cognizant to stimuli without response); immobile (not cognizant and lying on back with partial limb paralysis); paralyzed (not cognizant, on back with slight muscle twitch, and slow gut pulse); and dead (no movement).

### 2.4. High-Performance Liquid Chromatography and Mass Spectroscopy

For Test 2, 0.5 mL of blood was drawn from the marginal ear vein at 0, 6, 24, 36, 48, and 72 hours post-ivermectin injection. This blood was centrifuged at low speed, and the whole blood sample was frozen to −52°C and shipped on dry ice to Case Western Reserve University Proteomics Core facility, Cleveland, Ohio, for testing. The ivermectin concentration was analyzed using a modified method reported previously [13]. In brief, each sample was thawed and 50 microliters of supernatant was mixed with 500 microliters of 3 : 1 v/v methanol : acetonitrile, vortexed for 20 seconds, and incubated at 4°C for 30 minutes. The solution was centrifuged at 16,000 *g* for 20 minutes, and 500 microliters of supernatant was transferred to a clean tube and speed vacuumed to dryness. It was then reconstituted with 50 microliters of reconstitute solvent (0.5 mM ammonium formate; 0.1% formic acid in 50% methanol) and centrifuged at 16,000 *g* for 20 minutes. Five microliters were used for high-performance liquid chromatography (HPLC) and mass spectrometry (MS). The chromatography was performed with a reversed-phase C18 column (Atlantis dC18 column, 50 × 2.1 mm, 3 *µ*m, Waters). Ivermectin was separated from blood endogenous components using 10% 0.5 mM ammonium formate containing 0.1% formic acid in the isocratic mode at 0.2 ml/min. The column was set at 35°C. Ivermectin was detected by a Thermo Scientific TSQ Quantum Ultra with HESI-II probe using ESI positive ionization mode; spray voltage of 3000 V; capillary temperature of 200°C; vaporizer temperature of 300°C; sheath gas pressure of 40; aux gas pressure 10; skimmer offset 10 V; SRM setup: Q1: 0.7 FWHM, Q3: 0.7 FWHM, and Q2: 1.5 mTorr (Ar); scan width: 0.002 m/z; scan time at 0.02 s.

### 2.5. *Cimex lectularius* Bacterial Gut Cultures

Bed bugs from Test 2 had their gut contents cultured after the morbidity and mortality was recorded on day 64 after ivermectin injection. The healthiest appearing adults were used first, and if no adults were alive, then the healthiest appearing nymphs were used. Bed bugs were surface disinfected to minimize external contaminants by twice vortexing the insects for one minute in a sterile saline solution and pipetting off the fluid. Insects were then exposed to benzalkonium chloride for 1 minute and rinsed again in sterile saline. Rinsates (0.1 mL) from the last washing were spread onto blood agar culture plates to assess the disinfection protocol and found to reproduce no colonies after 72 hours. Bed bugs were thoroughly crushed with a sterile glass rod in 1 ml sterile saline. Serial dilutions were prepared at 10^0^, 10^1^, and 10^2^, and 0.1 ml was spread onto blood agar culture plates (Sigma-Aldrich Inc.) with 2 plates per dilution. Plates were incubated at 25°C for 3 days and then photographed.

### 2.6. Data Analysis

Comparison of proportions using the “*N* − 1” chi-squared test was used to assess for differences between bed bug morbidity and mortality for those harmed for females, males, and nymphs for each feeding time after injection compared to the control group (Time 0, before injection feeding). Comparison of proportions was used to determine differences in nymphal molting and the number of eggs laid for each feeding time after injection compared to the control group. We used ANOVA to compare group differences in bed bug mortality and incapacitation rates between females, males, and nymphs for feeding times after injection.

## 3. Results

The morbidity and mortality results are summarized in Tables 1 and 2 (Tests 1 and 2).

### 3.1. Mortality

Both morbidity and mortality were noted in the 6-hour post-ivermectin injection feeding group in Test 1. After 45 days, the mortality rate was 0/30 (0%), 2/30 (7%), 8/30 (27%), 7/30 (23%), 8/30 (27%), 12/30 (40%), 10/30 (30%), 8/30 (27%), 5/30 (17%), 2/30 (7%), 1/30 (3%), and 3/30 (10%) for the feedings done at 0, 1, 6, 12, 24, 36, 48, 72, 96, 120, 144, and 168 hours, respectively. Peak bed bug mortality occurred at 36 hours after ivermectin injection. The mortality rates for bed bugs fed 6–96 hour after ivermectin injection were significantly different from controls (*p* < 0.05).

Test 2 ivermectin levels in the blood were 0, 2.1, 8.3, 10.4, 12.4, and 18.3 ng/mL for 0, 6, 24, 36, 48, and 72 hours after injection, respectively. The Test 2 day 64 mortality rate was 6/20 (30%), 5/20 (25%), 9/21 (43%), 9/20 (45%), 4/20 (20%), 12/20 (60%), 8/20 (40%), 8/20 (40%), 10/31 (48%), 8/20 (40%), 10/20 (50%), and 8/20 (40%) for the feedings done at 0, 1, 6, 12, 24, 36, 48, 72, 96, 120, 144, and 168 hours, respectively, demonstrating a dose-dependent effect of ivermectin. Only the 36-hour post-ivermectin injection feeding group mortality was significantly different from that of the control group (*p* < 0.05). There was unexpectedly high mortality in all the adult females after feeding making it more difficult to ascertain differences between the experimental groups with the controls.

Adult *C. lectularius* from Tests 1 and 2 showed the highest mortality when fed between 6 and 48 hours after ivermectin injection. Nymph *C. lectularius* from Tests 1 and 2 showed the highest mortality when fed between 12 and 72 hours after ivermectin injection.

### 3.2. Incapacitation Rate

**Figure 1:**
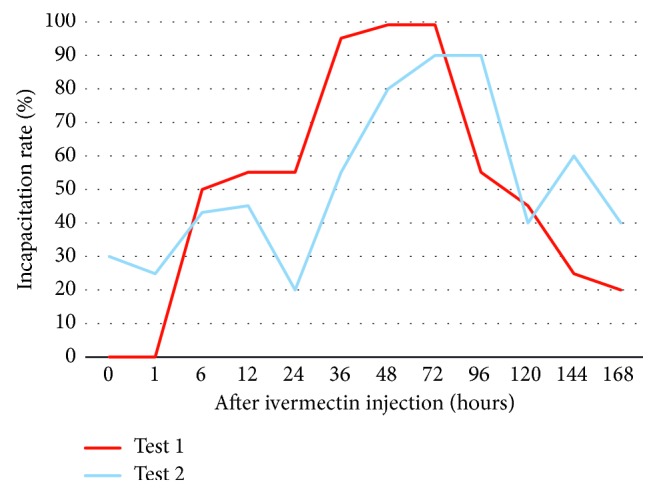
Bed bug incapacitation rates after feeding on a rabbit injected with 0.3 mg/kg of ivermectin (Test 1 and Test 2).

The incapacitation rate, or the number of insects that were harmed, is the number of bed bugs that were categorized as reactive, immobile, paralyzed, and dead over the number of healthy insects. The Test 1 day 45 incapacitation rate was 0%, 0%, 50%, 55%, 55%, 95%, 100%, 100%, 55%, 45%, 25%, and 20% for the feedings done at 0, 1, 6, 12, 24, 36, 48, 72, 96, 120, 144, and 168 hours, respectively. Peak bed bug harm occurred in the groups fed 36–72 hours after ivermectin injection. All bed bugs that fed after 6 hours after ivermectin injection had significantly more harm than controls (*p* < 0.05). In Test 2 after 64 days, the overall incapacitation rate was 30%, 25%, 43%, 45%, 20%, 60%, 80%, 90%, 90%, 40%, 60%, and 40% for the feedings done at 0, 1, 6, 12, 24, 36, 48, 72, 96, 120, 144, and 168 hours, respectively. Peak bed bug harm occurred in the groups fed 36–96 hours after ivermectin injection where there was significantly more harm than controls (*p* < 0.05).  Figure 1 shows the incapacitation rates of Test 1 and Test 2 in hours after ivermectin feeding.

### 3.3. Insect Paralysis

Ivermectin causes insect paralysis in a dose-dependent manner. However, bed bugs in the same feeding cohort had different responses to the drug after feeding. Some insects that fed on ivermectin-containing blood exhibited no response to light, painful stimuli, sound, and odor, with no apparent cardiac, enteric, or respiratory function that lasted a few weeks. Two males and one female in the 48-hour postinjection feeding cohort were torpid in this state for 21 days before regaining some life functions. In the 72-hour cohort, two males experienced torpor, one experienced some recovery while the other died.

### 3.4. Refeeding

Test 2 bed bugs were offered blood meals at 16 and 31 days after feeding on the rabbit. Controls and 1- and 6-hour postinjection cohorts fed normally. Some bed bugs, especially those fed 36–72 hours after injection, had difficulty refeeding, and no insects digested their blood meals. It was observed that the cibarial pump, esophagus, and first ventriculus seemed functional, and those that succeeded in skin penetration could not draw blood. Once they had withdrawn, there was no postfeeding defecation indicating a successful feeding. A number of these insects exhibited no control of the maxillary stylus and were physically unable to penetrate the skin. Either the stylus was distally bent, making penetration impossible or force of penetration caused it to loop out between the labrum and maxillary lobe. This led to repeated efforts to feed. Such behaviors as repeated beak grooming, elevated head positions attempting to move the beak into position, running in random circles, and backing up, all were indicative of distress. In the 36-hour postinjection cohort, 15% of the insects attempted to feed and failed; the 48-hour cohort saw no feeding; the 72-hour cohort tried to feed but could not; the 96-hour cohort was mostly nonresponsive and sessile, although a few bed bugs tried to feed and failed; the 120-hour adult cohort did not feed and 20% of nymphs tried but failed; the 144-hour cohort had some nymphs interested in feeding; and the 168-hour cohort initially exhibited disinterest but later all insects fed normally.

### 3.5. Fecundity

Ivermectin did not affect late-stage preformed eggs in female ovarioles. These eggs were laid and hatched into 1^st^ instar nymphs. Females exposed to sublethal ivermectin doses were often made infertile if oocytes were being formed in the germarium at the time of feeding. Eggs either never developed or were laid without yolk. The few first instar nymphs engendered by sublethal ivermectin-treated females that succeeded to hatch did not appear to be grossly affected and developed into adults.

Ivermectin had a dramatic and long-term decrease in both the number of eggs that were laid and the resulting new instars. In Test 1, there were >50 eggs laid in the control group, and only 5 eggs laid for all other feeding cohorts. In Test 2, there were >50 eggs laid in the control group, and 45, 23, 12, 0, 13, 0, 10, 9, 3, 10, and 27 eggs laid in the 1, 6, 12, 24, 36, 48, 72, 96, 120, 144, and 168 hours after ivermectin injection groups.

### 3.6. Molting

**Figure 2:**
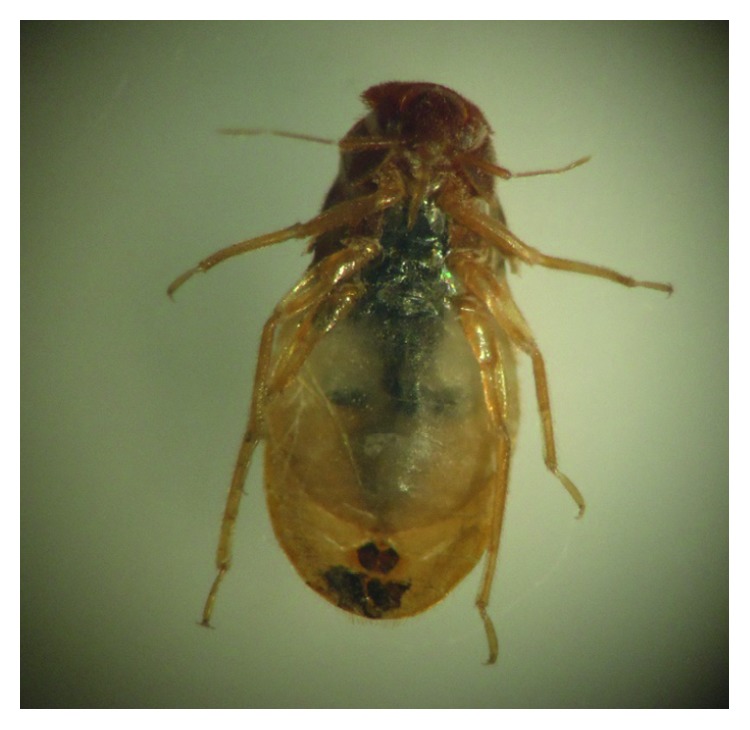
Nymph showing incomplete molt after taking an ivermectin blood meal.

Nymphal molting was significantly reduced for bed bugs that fed between 6 and 168 hours after injection (Figure 2). For Test 2, all nymphs were offered two blood meals and thus the opportunity to molt twice. By the end of the 64-day observation period for Test 2, high numbers of exuviae were present in the controls because 5^th^ instar females matured during the test period and engendered many 1^st^ and 2^nd^ instar nymphs. However, all Test 2 postinjection cohorts experienced suppression of nymphal development at day 64.

### 3.7. *Cimex lectularius* Gut Cultures

**Figure 3:**
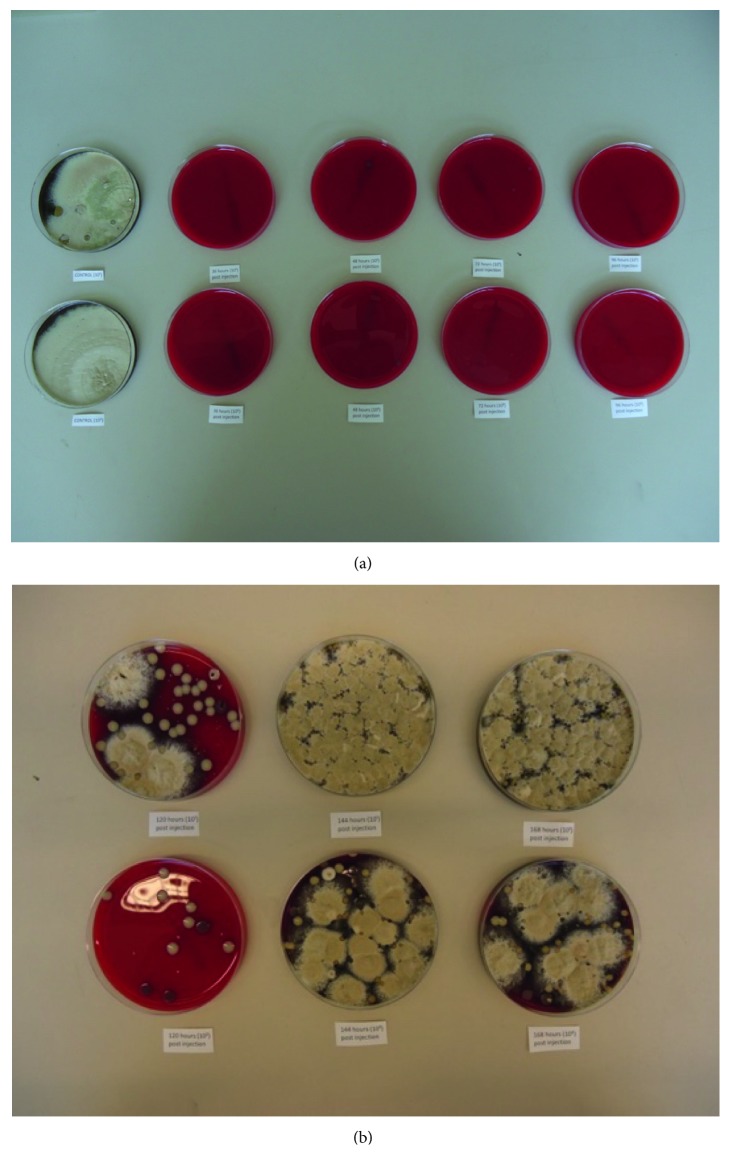
Agar plates of bed bug gut cultures from Test 2 (a) nymphs and (b) adults >64 days after feeding on the rabbit observed at 72 hours.

We found that bed bugs that fed on the rabbit between 36 and 96 hours after ivermectin injection had no microbial growth on blood agar plates. After 96 hour, bacterial growth was again observed. The growth plates are shown in Figure 3.

## 4. Discussion

We show that bed bugs which take an *in vivo* blood meal containing ivermectin suffer dose-dependent toxicity including death, reductions in fecundity, inability and disinterest in refeeding, and molting. Ivermectin blood concentrations of ∼2 ng/mL in the rabbit were required to see changes in bed bug fecundity, and concentrations of at least 8 ng/mL were required before other significant bed bug toxicity was observed. Our results are congruent with reports investigating ivermectin and bed bugs where morbidity and mortality was noted between 2.5 and 25 ng/mL using an *in vitro* feeding system [9, 10]. Ivermectin also alters the *C. lectularius* gut microbiome—the implications of which require further investigation.

There are limited *in vivo* data demonstrating the toxicity of ivermectin against bed bugs and no studies where ivermectin blood levels were measured. A small group of bed bugs were fed on mice after receiving high doses of ivermectin intraperitoneally, which resulted in high insect morbidity and mortality [8]. Bed bugs were also allowed to feed on four human volunteers given 0.2 mg/kg of oral ivermectin, which caused insect morbidity and mortality in a dose-dependent manner [8]. Limitations of these studies include that only a few bed bugs fed on each human subject, the insects were observed for less than a month, not all life stages were evaluated, the bed bugs were fed soon after the administration of ivermectin, and bed bugs were only reported as dead or harmed and did not account for other manifestations of morbidity such as reductions in fecundity or the ability to refeed.

In our experiments, we found that peak bed bug morbidity and mortality occurred when insects fed 36 to 72–96 hours after ivermectin injection. We were unable to obtain blood samples after 72 hours after ivermectin injection, and we are unsure if the peak serum concentration of ivermectin occurred between the 48–72 hour sample or after the 72-hour sample. Based on our bed bug morbidity and mortality results and the previously reported pharmacokinetics of subcutaneous ivermectin in a rabbit, we suspect the peak plasma concentration in the rabbit occurred around 72 hours after injection [14, 15]. The pharmacokinetics of subcutaneously administered ivermectin in a rabbit does not mirror the pharmacokinetics of orally administered ivermectin in humans which reaches a peak plasma concentration in about four hours [11].

There have not been any human clinical trials using ivermectin for bed bug control. Our data show that ivermectin may not need to kill all bed bugs in the population in order to eliminate an infestation because the ivermectin greatly reduces fecundity, prevents nymphal molting, and inhibits insect refeeding. Ivermectin could potentially be used as an adjunctive therapy for humans with bed bugs along with traditional integrated pest management (IPM) practices.

## 5. Conclusions

Ivermectin causes dose-dependent toxicity in *C. lectularius* when fed on a rabbit injected with ivermectin. Ivermectin blood levels of ∼2 ng/mL cause changes in bed bug fecundity, and levels of >8 ng/mL are required for significant bed bug mortality. Ivermectin causes alterations in the bed bug gut microbiome.

## Figures and Tables

**Table 1:** Test 1 data with incapacitation rate for each life stage, exuviae, and eggs at day 45 for each group fed at different hours after ivermectin injection.

Hours	Females (*n*=5)	Males (*n*=5)	Nymphs (*n*=10)	% dead or harmed	Mortality rate (%)	Exuviae	Eggs	Notes
0	5 healthy	5 healthy	10 healthy	0	0	>50	>50	Healthy
1	5 healthy	5 healthy	10 healthy	0 *p*=1.0	0 *p*=1.0	>50	>50	
6	5 dead	2 dead, 2 harmed, 1 healthy	1 dead, 9 healthy	50 *p* < 0.001	40 *p*=0.002	0	0	2 torpid nymphs appeared to recover health
12	5 dead	1 dead, 2 harmed, 2 healthy	2 dead, 1 harmed, 7 healthy	55 *p* < 0.001	40 *p*=0.002	0	0	Early adult deaths
24	2 dead, 3 healthy	2 dead, 1 harmed, 2 healthy	4 dead, 2 harmed, 4 healthy	55 *p* < 0.001	30 *p*=0.009	0	0	Early nymph and adult deaths
36	5 dead, 1 healthy	2 dead, 2 harmed, 1 healthy	5 dead, 5 harmed	95 *p* < 0.001	60 *p* < 0.001	1	0	All insects quickly immobile and no blood digestion
48	5 dead	3 dead, 2 harmed	3 dead, 7 harmed	100 *p* < 0.001	55 *p* < 0.001	0	0	High level of harm during first 10 days after feeding, no blood digestion
72	2 dead, 3 harmed	5 harmed	6 dead, 4 harmed	100 *p* < 0.001	40 *p*=0.002	0	0	Some harmed insects appeared to recover health, no blood digestion
96	1 dead, 4 healthy	1 dead, 2 harmed, 2 healthy	3 dead, 4 harmed, 3 healthy	55 *p* < 0.001	25 *p*=0.02	0	0	Some harmed insects appeared to recover health
120	3 harmed, 2 healthy	2 harmed, 3 healthy	2 dead, 2 harmed, 6 healthy	45 *p*=0.008	10 *p*=0.15	4	5	Most insects appeared harmed and some appeared to recover health
144	1 harmed, 4 healthy	1 harmed, 4 healthy	1 dead, 2 harmed, 7 healthy	25 *p*=0.02	5 *p*=0.32	0	0	Most population appeared to recover health
168	1 harmed, 4 healthy	1 dead, 4 healthy	2 dead, 8 healthy	20 *p*=0.04	15 *p*=0.08	0	0	Population appears mostly healthy

**Table 2:** Test 2 data with each life stage recorded as dead (D), incapacitated (I), or healthy (H) at day 64 after feeding. Also recorded are the breath test, feeding 1 test, feeding 2 test, exuviae, and eggs at day 64 for each group fed hours (Hr) after ivermectin injection.

Hours	IVM level (ng/mL)	Females (*n*=5)	Males (*n*=5)	Nymphs (*n*=10)	% dead or harmed	Mortality rate (%)	Breath test day 14	Feeding 1 on day 16	Feeding 2 on day 31	Exuviae	Eggs
0	0	5 D	1 D, 4 H	10 H	30	30	Normal	Normal	Normal	>50	>50 (>50 new nymphs)
1		5 D	5 H	10 H	25 (*p*=0.73)	25 (*p*=0.73)	Normal	Normal	Normal	15	45 (27 new nymphs)
6	2.1	5 D	2 D, 3H	2 D^*∗*^, 9H	43 (*p*=0.39)	43 (*p*=0.39)	Low interest	None	Normal	11	23 (13 new nymphs)
12		5 D	2D, 3 H	2 D, 8 H	45 (*p*=0.33)	45 (*p*=0.33)	Low interest	Low interest	Low interest	9	12 (9 new nymphs)
24	8.3	1 D, 4 H	1 D, 4 H	2 D, 8 H	20 (*p*=0.47)	20 (*p*=0.47)	Low and no interest	Low and no interest	Normal	4	0
36		5 D	5 D	2 D, 8 H	55 (*p*=0.11)	55 (*p*=0.11)	Low and no interest	Low and no interest	Failed feeding attempt and low interest	3	13 (8 new nymphs)
48	12.4	5 D	3 D, 2 I	6 I, 4 H	80 (*p*=0.002)	40 (*p*=0.51)	No interest	No interest	No interest	3	0
72	18.3	3 D, 1 I, 1 H	4 D, 1 I	1 D, 8 I, 1 H	90 (*p* < 0.001)	40 (*p*=0.51)	Low and no interest	None	Failed feeding attempt	7	10 (4 new nymphs)
96		4 D, I, H	4 D, 1 I	2 D^*∗*^, 9 I	90 (*p* < 0.001)	50 (*p*=0.2)	No interest	No interest	Failed feeding attempt and low interest	5	9 (8 new nymphs)
120		4 D, 1 H	3 D, 2 H	1 D, 9 H	40 (*p*=0.51)	40 (*p*=0.51)	No interest	No interest	Failed feeding attempt and low interest	6	3 (0 new nymphs)
144		5 D	3 D, 2 I	2 D, 8 H	60 (*p*=0.06)	50 (*p*=0.2)	No interest	Failed feeding attempt and low interest	Failed feeding attempt and low interest	5	10 (9 new nymphs)
168		5 D	2 D, 3 H	1 D, 9 H	40 (*p*=0.51)	40 (*p*=0.51)	Normal	Normal	Failed feeding attempt and low interest	5	27 (25 new nymphs)

^*∗*^11 nymphs were fed.

## Data Availability

The data used to support the findings of this study are available from the corresponding author upon request.
